# SCARA5 induced ferroptosis to effect ESCC proliferation and metastasis by combining with Ferritin light chain

**DOI:** 10.1186/s12885-022-10414-9

**Published:** 2022-12-13

**Authors:** Yanqun Liu, Rong Xiong, Ting Xiao, Li Xiong, Jialin Wu, Junfeng Li, Gang Feng, Guiqin Song, Kang Liu

**Affiliations:** 1 Institute of Tissue Engineering and Stem Cells, The Second Clinical Medical College of North Sichuan Medical College, Nanchong Central Hospital, Nanchong, 637000 China; 2 Department of Cell Biology and Genetics, North Sichuan Medical College, Nanchong, 637100 China; 3 Department of Laboratory Medicine, Sichuan Chengdu Chengfei Hospital, Chengdu, 610092 China

**Keywords:** ESCC, SCARA5, Ferritin light chain, Ferroptosis

## Abstract

**Background:**

Esophageal squamous cell carcinoma (ESCC) remains one of the most lethal cancers worldwide accompany with an extremely poor prognosis. Therefore, this study aims to screen for new molecules affecting ESCC and explore their mechanisms of action to provide ideas for targeted therapies for ESCC.

**Methods:**

Firstly, we screened out the membrane protein SCARA5 by high-throughput sequencing of the ESCC patient tissues, and RT-qPCR and WB were used to verify the differential expression of SCARA5 in esophageal cell lines, and IHC analyzed the expression localization of SCARA5 in ESCC tissue. Then, flow cytometry, wound healing assay, Transwell assay and CCK-8 assay were used to explore the effects of SCARA5 on cell cycle, migration and invasion as well as cell proliferation activity of esophageal squamous carcinoma cells. Meanwhile, transmission electron microscopy was used to detect changes in cellular mitochondrial morphology, and flow cytometry were used to detect changes in intracellular reactive oxygen metabolism, and immunofluorescence and flow cytometry were used to detect changes in intracellular Fe^2+^. Mechanistically, co-immunoprecipitation was used to detect whether SCARA5 binds to ferritin light chain, and ferroptosis-related protein expression was detected by WB. Finally, the tumor xenograft model was applied to validation the role of SCARA5 tumor growth inhibition in vivo.

**Results:**

We found that SCARA5 was aberrantly decreased in ESCC tissues and cell lines. Furthermore, we confirmed that SCARA5 suppressed the cell cycle, metastasis and invasion of ESCC cells. Meanwhile, we also found that overexpression of SCARA5 caused changes in mitochondrial morphology, accumulation of intracellular reactive oxygen species and increased intracellular Fe^2+^ in ESCC cells, which induced ferroptosis in ESCC cells. Mechanically, we validated that SCARA5 combined with ferritin light chain and increased intracellular Fe^2+^. As well as, overexpression *SCARA5* induced ferroptosis by increasing ferritin light chain in nude mice subcutaneous tumors and inhibited the growth of nude mice subcutaneous tumors.

**Conclusion:**

Collectively, our findings demonstrated that *SCARA5* suppressed the proliferation and metastasis of ESCC by triggering ferroptosis through combining with ferritin light chain.

**Supplementary Information:**

The online version contains supplementary material available at 10.1186/s12885-022-10414-9.

## Introduction

Esophageal cancer is one of the most common digestive tract malignancies. Its incidence ranks the seventh and mortality ranks the sixth among all malignancies [1], according to Global Cancer Statistics, there were approximately 604,000 new cases and 544,000 deaths of esophageal cancer in 2020. As the most common histologic subtypes of esophageal cancer, ESCC had the sixth highest incidence and the fifth highest mortality rate of malignant tumors in China [2, 3]. Although the diagnostic and therapied methods constantly improved in recent years, the prognosis of most patients remains poor, with the 5-year overall survival rate of only 15% ~ 25% [4]. Therefore, there is an urgent need to characterize further mechanism of effecting the progression of ESCC for more efficient anti-tumor treatment.

The scavenger receptor family, a superfamily of membrane-bound receptors, contain five family members forming homotrimers of type II transmembrane proteins on the cell surface [5]. Many biomolecules such as modified lipoproteins, lipids, polyribonucleic acids and polysaccharides can bind this membrane receptor, which is a macrophage receptor with a collagen structural domain [6]. Unlike other family members that are found primarily in macrophages, scavenger receptor protein 5 (*SCARA5*), located on chromosome 8p21, is widely expressed in a variety of human tissues, including bladder, ovary, kidney, and skin [7]. SCARA5 has been reported to act as a tumor suppressor in many cancers [6, 8–12]. For example, Huang and Ulker et al. respectively reported that the expression of *SCARA5* was specifically decreased in hepatocellular carcinoma and breast cancer due to promoter methylation [6, 8]. Meanwhile, Liu et al. found that CNS5 and SPAG5 could affect hepatocellular carcinoma progression by mediating ubiquitination of β-catenin to reduce the expression of *SCARA5* [9, 10]. Moreover, Chen and Liu et al. also discovered that the aberrant expression of *SCARA5* in thyroid cancer and lung cancer impacts cellular invasion and migration [11, 12]. What’s more, Li et al. reported that SCARA5 could act as ferritin receptor to facilitate intracellular iron increasing and promote the kidney development [13]. However, it is currently unknown whether SCARA5 binds to ferritin to affect the progression of ESCC.

Iron is considered an essential mineral involved in a wide variety of biological processes in organisms such as oxygen transport, enzyme-catalyzed reactions, aerobic respiration, lipid peroxidation, and DNA and RNA synthesis [14, 15]. However, excessive iron can feed the Fenton reaction to generate unquenchable amounts of free radicals that lead to the accumulation of reactive oxygen species and thereby induce ferroptosis, resulting in a certain toxic effect [16]. Ferroptosis is an iron-dependent form of regulated cell death characterized by the accumulation of intracellular reactive oxygen species, and it has recently been reported as a potential direction for the treatment of a variety of tumors [17, 18]. Morphologically, distinct from traditional cell death types such as apoptosis and necrosis, ferroptosis is mainly characterized by reduced mitochondrial volume, reduced cristae, and increased membrane density [19]. In terms of biochemical characteristics, ferroptosis is mainly manifested as accumulation of intracellular iron ions and reactive oxygen species and inhibition of the cystine/glutamate transport protein system [20]. Mechanistically, ferroptosis can be initiated by exogenous or transporter protein dependent pathways as well as endogenous or enzyme regulated pathways [21]. Ferritin is an intracellular iron storage protein composed of two subunits, including ferritin light chain and ferritin heavy chain, which is mainly involved in intracellular iron storage and utilization, and protects cells from damage caused by imbalance of iron metabolism [22]. It has been reported that ferritin is a marker for cellular ferroptosis and is involved in the ferroptosis in a variety of tumors [23, 24], such as pancreatic cancer [25], hepatocellular carcinoma [26], lung adenocarcinoma [27], and glioma [28], which provides a new direction for the treatment of cancer. In addition, it has been reported that SCARA5 binding to ferritin light chain causes increased cell death in cervical cancer cells [29]. However, it is unclear whether ferritin is involved in iron ion transport in ESCC cells to induce ferroptosis.

In this study, we first sequenced the whole transcriptome of 6 pairs of ESCC tissues and adjacent tissues to screen out the tumor suppressor gene *SCARA5* that is low expressed in ESCC. We then investigated the effect of overexpression of SCARA5 on the cell cycle, invasion and migration of ESCC cells, and explored whether overexpression of *SCARA5* induced ferroptosis in ESCC cells. Finally, we further demonstrated that SCARA5 increased accumulation of Fe^2+^ and reactive oxygen species in ESCC cells through binding to ferritin light chain and thereby induced ferroptosis in ESCC cells.

## Methods

### Ethics

The 6 pairs of human ESCC tissues and their matched adjacent tissues were collected for high-throughput sequencing from Nanchong Central Hospital (Nanchong, China), and the data was also available at https://www.ncbi.nlm.nih.gov/geo/query/acc.cgi?acc=GSE205121. This project was supported by the Ethics Committee of Nanchong Central Hospital [2019095]. All patients did not receive treatment and signed an informed consent form.

### ESCC cell lines and cell culture

The human ESCC cell lines KYSE30, KYSE410 were purchased from Procell Life Science &Technology (Wuhan, China), KYSE150 and TE-1 were purchased from GENECHEM (Shanghai, China), TE-11 and KYSE510 were purchased from Shanghai XuanYi Biotechnology Service Center (Shanghai, China), and the human normal esophageal epithelial cell line HEEC was purchased from Keygen Biotech (Jiangsu, China). All of them were incubated in RPMI-1640 Medium (Gibco, USA) with 10% fetal bovine serum (Biological Industries, USA) and 1% penicillin/streptomycin (Gibco, USA), and the human normal esophageal epithelial cell line HET-1A was cultured in DMEM Medium (Gibco, USA) with 10% fetal bovine serum and 1% penicillin/streptomycin and incubation with 5% CO2 at 37℃, which were purchased from Keygen Biotech (Jiangsu, China).

### Construction of plasmids, lentivirus and stable transfection into cell lines

The *SCARA5* coding sequence was inserted into the pcDNA3.1 vector (pcDNA3.1-SCARA5, SCARA5-OE vector). The stable transfection ESCC cell lines were constructed from GENECHEM CO., Ltd (Shanghai, China). pcDNA3.1-SCARA5 and SCARA5-OE vector were transfected using Lipofectamine™ 2000 (Invitrogen, Carlsbad, CA, USA).

### Quantitative real-time PCR analysis

Total RNA of these different cells was extracted by using Trizol Reagent (Vazyme, China). Then, total RNA was reverse transcribed into cDNA using the HiScript® III RT SuperMix (Vazyme, China) with the following temperature protocol: 37℃ for 15 min, 42℃ for 5 s. Quantitative real-time PCR (qPCR) was performed using the ChamQ Universal SYBR qPCR Master Mix (Vazyme, China). The primer sequences of key genes shown in the Supplementary Table 1.

### CCK8 assay

The stable cell lines TE-1 and KYSE150 with overexpression *SCARA5* in the logarithmic growth phase were seeded in a 96-well plate with the density of 3 × 10^3^ cells per well and incubated. Then, the CCK8 assay (KeyGEN BioTECH, Jiangsu, China) was used to measure the proliferation abilities of stable cell lines at 0 h, 24 h, 48 h, 72 h and 96 h, each well added 10% CCK8, incubated for 1 h at 37℃, and the absorbance at 450 nm were detected in microplate reader (BioTek Instrument Inc., Winooski, VT, USA). Cell viability was measured after cells were treated with Ferrostain-1 (1 μM), Erastin (10 μM), Necrostain-1 (10 μM), Z-VAD-FMK (10 μM) and 3-MA (60 μM). All experiments were performed in quadruplicate.

### EdU incorporation assay

According to the manufacturers’ instruction, the 2 × 10^4^ cells TE-1 and KYSE150 cells were seeded in 24-well plates to determine the number of proliferation cells with an Edu kit (BeyoClick™ EdU-488, Beyotime).

### Flow cytometric analysis of percentage of cell death

The TE-1 and KYSE150 cells were seeded in 24-well plates for 24 h, and the Annexin V-FITC/PI Apoptosis Detection Kit (#A211‑02, Vazyme) was utilized to detect the percentage of cell death according to the manufacturers’ instructions.

### Cell cycle analysis

The stable cell lines TE-1 and KYSE150 cells were seeded in a 6-well plate with the density of 5 × 10^5^. After incubation 24 h, these cells were harvested and washed twice with cold PBS and then fixed with 75% cold ethanol overnight at -20℃. Subsequently, the fixed cells were centrifuged and washed twice with cold PBS and resuspended in binding buffer (Cell Cycle Analysis Kit, 4A, Biotech) containing 100 μg/mL propidium iodide and 200 μg/mL Rnase A for 30 min at 37℃ in the dark. Stained cells were collected by a FACSCalibur flow cytometer (BD Biosciences), and the data was analyzed by Flow Jo software (BD Biosciences, USA).

### Wound healing assay

The stable cell lines TE-1 and KYSE150 were plated into six-well plates at 4 × 10^5^cells/well and grown to confluency. And two separate parallel wounds were generated by scratching the cell layer with a 10 μL plastic pipette tip. The numbers of migrated cells were observed and imaged under microscope (ECLIPSE TS100, Nikon) at the time points 0 h and 24 h. These experiments were performed in triplicate.

### Transwell assay

The migration or invasion assays were performed using polycarbonate Transwell filter chambers (8 μm pore size; Corning Inc.) and the inserts were coated with or without Matrigel® (BD Biosciences). A total of 2 × 10^4^ cells were seeded in the upper transwell chamber and 1 × 10^5^ cells were added in the top chamber containing Matrigel with 100μL serum-free medium, whereas the bottom chamber was added with 500μL medium containing 10% serum. After being cultured for 48 h, the migrated cells on the lower membrane were stained with 0.1% crystal violet (KGA229, Keygen Biotech) and counted.

### Iron assay

Intracellular chelable iron was determined using the fluorescent indicator Phen green SK (#P14312), the fluorescence of which is quenched by iron. 5 × 10^4^ cells were inoculated into a 24-well plate respectively, and climbing slides were added, and then transfection reagent was added after incubating at 37 °C for 24 h. After 48 h of transfection, the culture medium was discarded, the slides were washed with PBS three times, and the 20 μM Phen green SK probe was added with incubated at 37 °C for 20 min, and the slides were washed with PBS three times, and then photographed by ordinary fluorescence microscope. Meanwhile, 3 × 10^5^ cells were inoculated into a 6-well plate, incubated at 37 °C for 24 h, and then added transfection reagent, respectively. After 48 h of transfection, the cells were collected and added 20 μM phen green SK probe, incubated at 37 °C for 20 min, washed with PBS three times. The intracellular chelable iron was measured by a FACSCalibur flow cytometer (BD Biosciences, Franklin Lakes, NJ).

### Measurement of ROS

The peroxide-sensitive fluorescent probe DCFH-DA (#CA1410) was used to detect intracellular total ROS and the peroxide-sensitive fluorescent probe C11-BODIPY (#D3861) was used to detect the lipid ROS according to the manufacturer’s instruction. 3 × 10^5^ cells were inoculated into a 6-well plate respectively, incubated at 37 °C for 24 h, and then added transfection reagent. After 48 h of transfection, the cells were collected and incubated at 37℃ for 30 min with 10 μM DCFH-DA probe and incubated at 37℃ for 20 min with 5uM C11-BODIPY probe. Cells were then washed with PBS three times and were analyzed by flow cytometry (BD Biosciences, Franklin Lakes, NJ).

### Malondialdehyde (MDA) assay

MDA, as a major indicator of lipid peroxidation, was detected using MDA Assay Kit (#A003-4–1) according to the manufacturer’s instructions, which was purchased from Nanjing Jiancheng. Protein concentration was assayed using a Beyotime BCA Protein Assay Kit according to the manufacturer’s instructions.

### GSH assay

The total quantities of glutathione were measured using a GSH Assay Kit (#KTB1600), which was purchased from Abbkine. Then, the GSH of each of group was detected by referring to the manufacturer’s instructions.

### Co-immunoprecipitation (Co-IP)

The cell pellets of ESCC cells TE-1 and KYSE150 were lysed, the protein concentration was determined by the BCA assay, and the expression levels of SCARA5 and FTL were detected. Then, each group was added 200ul of Protein A + G agarose beads, and added FTL antibody to the IP group, and rabbit IgG to the IgG group, and bonded for 2 h at 4 °C. Add cell lysate to each group and rotate overnight at 4 °C. Wash with PBS 3 times, add SDS loading buffer for elution. The elution proceeded with standard western blotting.

### Western blot analysis

Cells were harvested and then lysed with RIPA lysis buffer on ice. The BCA Protein Assay Kit was utilized to determine the protein concentration was determined using. Protein samples were separated on 10% or 12% SDS-PAGE gels and transferred to a nitrocellulose membrane. The membranes were cut prior to hybridization with antibodies during blotting. The membranes were blocked with 5% non-fat milk, and incubated overnight with primary antibody at 4℃. After washing with PBS, the membrane was incubated with secondary antibody at room temperature for 1 h. The protein bands were visualized by enhanced chemiluminescence reagents (Thermo Scientific). GAPDH and β-tubulin were used as a housekeeping control protein. Primary antibodies against SCARA5 (#ab118894), FTL (#ab69090), FTH1 (#ab75973, #ET1705-55), TFR1 (#136,800, Invitrogen).

### Immunohistochemistry

Expression and distribution of SCARA5 in 5 μm thick paraffin section of ESCC patients and adjacent precancerous tissues was detected by immunohistochemistry staining. Immunohistochemical staining was estimated on the basis of previous studies in our laboratory [30]. Firstly, sections were dewaxed, rehydrated and then antigen retrieval with 0.01 M sodium citrate buffer (pH 6.0). After sections were blocked with 3% hydrogen peroxide for 10 min, and incubated with 0.1% Triton X- 100 for 10 min, and then blocked with 3% BSA, followed by incubation with primary antibodies (37 °C, 2 h). Subsequently, the slides were detected with antirabbit HRP secondary antibody for 1 h at room temperature. Finally, the sections were developed with 3,3ʹ-diaminobenzidine tetrahydrochloride (DAB) and counterstained with hematoxylin. Meanwhile, detecting the expression of SCARA5/FTL/FTH1 in the subcutaneous tumor nude mice in the control and overexpression SCARA5 group by immunohistochemistry.

### In vivo tumor model

Male BALB/C mice (5-6 weeks old) were purchased from the Beijing Huafukang Biotechnology Co., Ltd. The stable overexpression SCARA5 or vector KYSE150 cells (1 × 10^6^ cells in 200μL PBS) were injected subcutaneously into the dorsal region of each mouse. The mice weight and tumor size were measured every three days after 4 days of injection. All the mice were sacrificed after 24 days. Tumor volume (mm^3^) was calculated as follows: volume = length × width^2^ × 0.5. All the tumor was placed in 4% paraformaldehyde and then embedded with paraffin. H&E staining was performed to evaluate the tissue morphology.

### Statistical analysis

GraphPad prism 8.0 was used to performed Statistical analysis. All data were presented as mean ± standard deviation (SD) of at least three independent experiments. One way analysis of variance (ANOVA) was used for comparison differences between groups, and the statistical significance of the difference of mean values between two group was determined using Student’s t test. Data were considered statistically significantly when *P* < 0.05.

## Results

### *SCARA5* expression is downregulated in ESCC

**Fig. 1:**
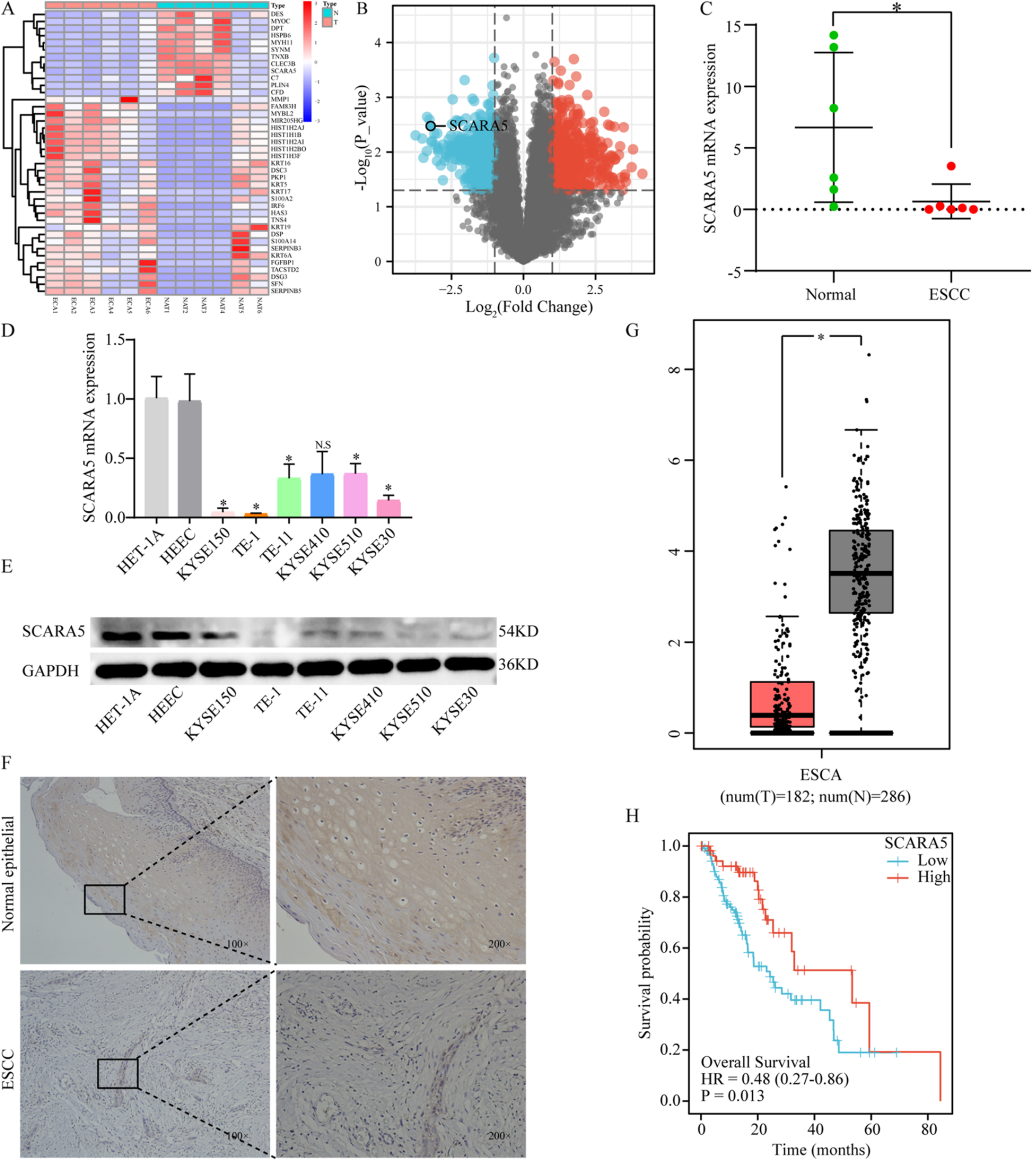
SCARA5 expression in ESCC tissues and cell lines. **a** Cluster Analysis Heatmap. **b** Difference Analysis Volcano Plot. **c** Sequencing results of SCARA5 in 6 pairs of esophageal squamous cell carcinoma tissues and adjacent tissues. **d** Relative SCARA5 mRNA expression in ESCC cells lines and the human normal esophageal epithelial cells line. **e** Relative SCARA5 protein expression in ESCC cells lines and the human normal esophageal epithelial cells line. **f** immunohistochemical staining of SCARA5 in ESCC tissues and para-carcinoma tissue. **g** Box plots showing SCARA5 gene expression levels in GEPIA database. **h** Survival analysis in the TCGA dataset. *, *P* < 0.05. The original blots/gels are presented in the Supplementary Material 3

First, differentially expressed genes were analyzed from the whole transcriptome sequencing results of 6 pairs of ESCC tissues and adjacent tissues. The heatmap showed obvious grouping and clustering (Fig. 1A), and the volcano map showed 614 up-regulated genes and 520 down-regulated genes with a threshold of |log2FC|> 1, *P* < 0.05 (Fig. 1B). Next, we screened six genes by querying the expression localization of differential genes and validated the expression in ESCC cells (Fig. S1), among which *SCARA5* was significantly attenuated in the sequencing results of ESCC (Fig. 1C). Then, the expression of SCARA5 was measured by RT-qPCR and Western blot in ESCC cell lines and normal esophageal epithelial cells (HEEC and HET-1A), the results showed that the expression of *SCARA5* was drastically reduced at mRNA and protein level in ESCC cells, especially in TE-1 and KYSE150 cells (Fig. 1D, E). Meanwhile, to further determine the expression of SCARA5 in ESCC tissues, we collected pathological sections of 20 patients to perform IHC analysis (Table 1), and the results exhibited that the intensity of SCARA5 immunostaining in the ESCC tissues was significantly decline compared with that in the matched non-cancerous tissues (Fig. 1F). Finally, combining the GEPIA and TCGA databases further verified the SCARA5 expression and prognosis, the results showed that the mRNA expression of SCARA5 in ESCA tissues was markedly attenuated compared with adjacent normal tissues (Fig. 1G) and patients with high SCARA5 expression had a longer survival time (Fig. 1H).

**Table 1:** Clinical information on 20 patients with ESCC

Characteristic	Case number	Rate ( %)
Age (years)		
< 60	3	15.0
≥ 60	17	85.0
Gender		
Female	7	35.0
Male	13	65.0
T stage		
T1	3	15.0
T2	5	25.0
T3	11	55.0
T4	1	5.0
N stage		
N0	10	50
N1	10	50
M stage		
M0	20	100

### Overexpression *SCARA5* depressed cell proliferation and increased the percentage of cell death

**Fig. 2:**
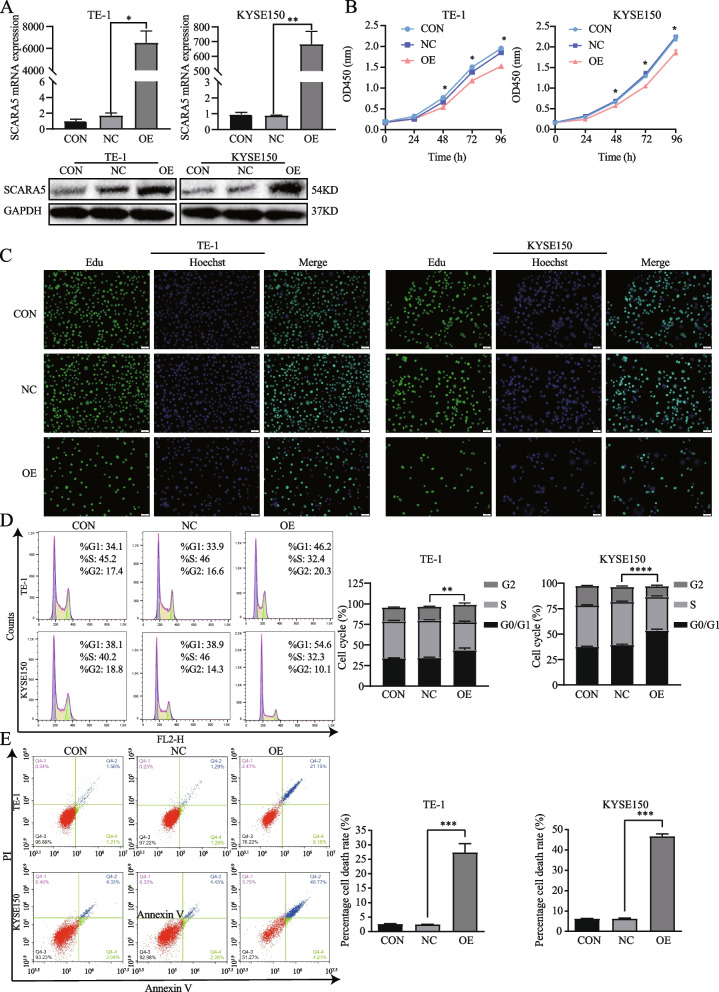
Overexpression SCARA5 inhibited ESCC proliferation ability and increased the cell death. **a** RT-qPCR and Western blot analysis of overexpression efficiency of SCARA5 in the corresponding cell lines. **b** Cell viability was evaluated at the indicated time points using CCK-8 assay. **c** Edu assay showed the proliferation of ESCC cells**.** d Cell cycle was examined by flow cytometric analysis. **e** Cell death was detected by flow cytometric analysis. *, *P* < 0.05; **, *P* < 0.01; ***, *P* < 0.001; ****, *P* < 0.0001. The original blots/gels are presented in the Supplementary Material 3

To explore the biological function of *SCARA5* in the ESCC cells, TE-1 and KYSE150 cells lines stable *SCARA5* overexpression were constructed by a lentiviral system. RT-qPCR and Western blot analysis were performed to confirm the overexpression efficiency of SCARA5. The results indicated the mRNA level and protein level of *SCARA5* was successfully overexpressed (Fig. 2A). Next, we firstly detected the proliferation activity of ESCC cells by CCK-8 assay. As shown in Fig. 2B, the cell viability of TE-1 and KYSE150 with stable overexpression SCARA5 was gradually reduced from 48 h onwards. In addition, to further observe the anti-proliferation effect of overexpression of SCARA5, the edu assay was applied. The number of Edu positive cells was significantly reduced in the overexpressed SCARA5 groups compared to the controls (Fig. 2C). Then, the effect of *SCARA5* on the cell cycle of TE-1 and KYSE150 cells was further analyzed, flow cytometric analysis showed that overexpression *SCARA5* led to a higher distribution of TE-1 and KYSE150 stable cells in G0/G1 phase versus the S and G2 phase (Fig. 2D), indicating that overexpression *SCARA5* effectively induced cell cycle arrest in G0/G1 phase in TE-1 and KYSE150 cells. Finally, to determine whether overexpression of SCARA5 increases cell death, we performed flow cytometry assays. The results indicated that the percentage of cell death was significantly increased in overexpressed SCARA5 groups (Fig. 2E). Taken together, these results suggested that *SCARA5* might inhibit ESCC cell proliferation ability and increased cell death.

### Overexpression of *SCARA5* suppresses the migration and invasion of ESCC cells

**Fig. 3:**
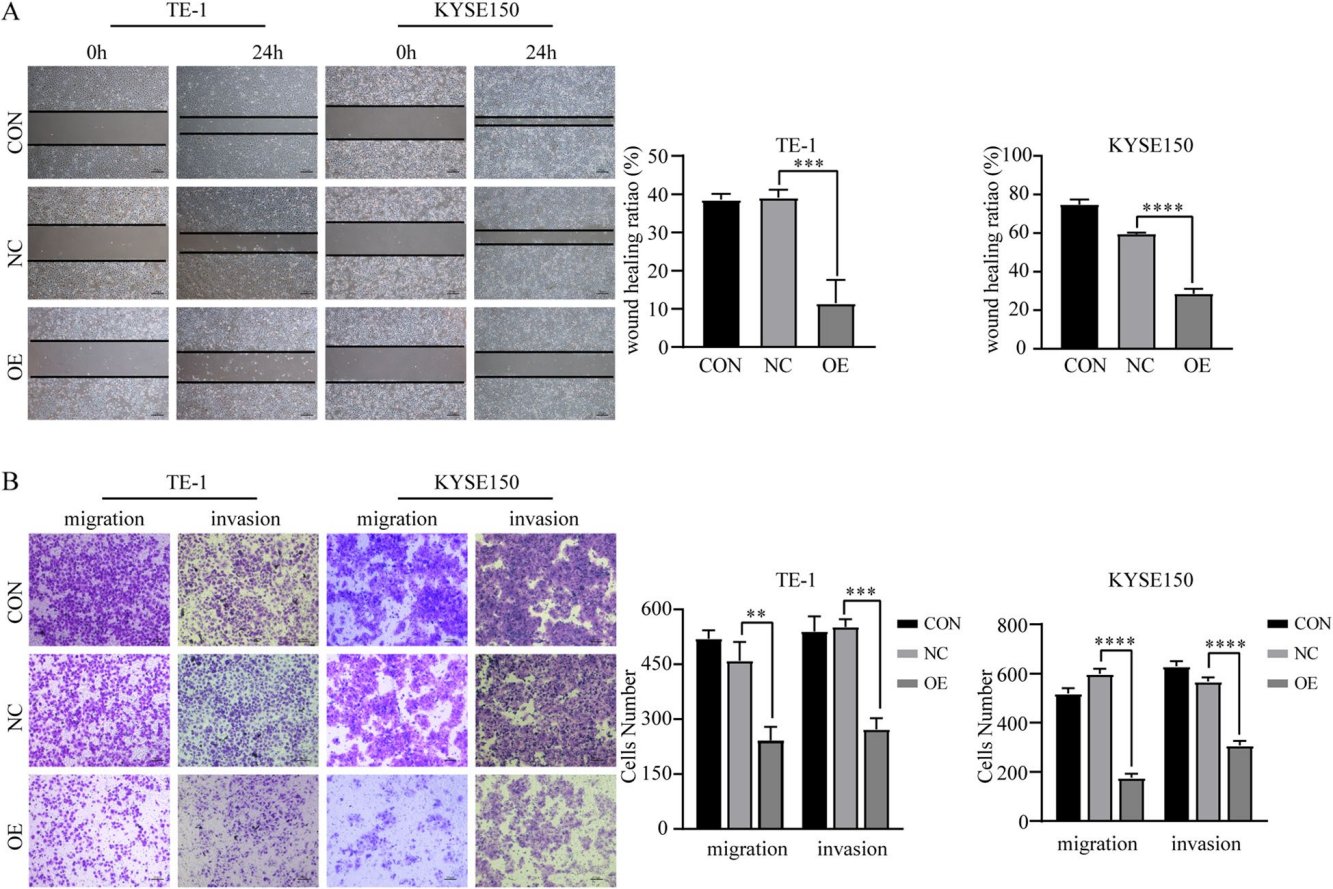
Overexpression SCARA5 inhibited ESCC cell migration and invasion.** a** Wound healing assay was used to determine the effect of SCARA5 overexpression on the migration ability of TE-1 and KYSE150, scale bar = 200 μm. **b** The effect of SCARA5 knockdown on the migration and invasion abilities of ESCC cells was evaluated by transwell assays, scale bar = 200 μm. **, *P* < 0.01; ***, *P* < 0.001; ****, *P* < 0.0001

To further examine the role of *SCARA5* in ESCC cells, the migratory and invasive capacity of the TE-1 and KYSE150 cells in which *SCARA5* was overexpressed was assessed by wound healing and transwell assays. The wound healing assay indicated that the overexpression of *SCARA5* markedly suppressed the migration ability of TE-1 and KYSE150 stable cells (Fig. 3A). Furthermore, transwell migration assay revealed that fewer cell migrated to the bottom of the chamber in TE-1 and KYSE150 cells with *SCARA5* overexpression, compared with control group cells (Fig. 3B). Consistently, transwell invasion assay also showed that the control groups had more cells invaded in the bottom of the chamber compared with the overexpression *SCARA5* group cells (Fig. 3B). Taken all, these results of above indicated that SCARA5 plays an important role in the migration and invasion of TE-1 and KYSE150 cells.

### Overexpression *SCARA5* induced ferroptosis in ESCC cells

**Fig. 4:**
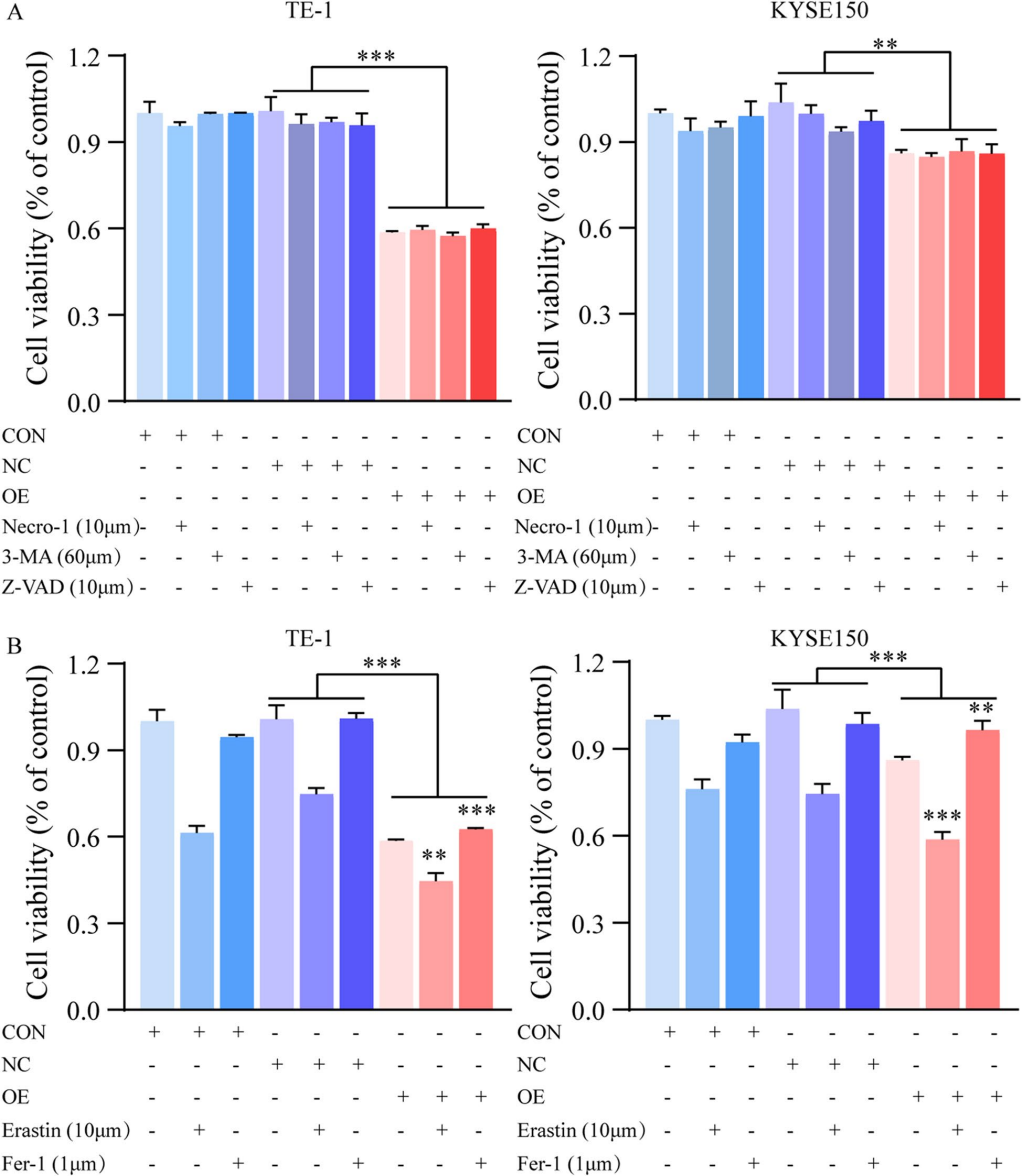
Overexpression SCARA5 induced ferroptosis in ESCC cells. **a** The effect of SCARA5 in combination with other cell death inhibitors on the cell viability of ESCC cells after the treatment for 24 h. **b** The effects of ferroptosis inhibitors and inducers on the activity of ESCC cells. **, *P* < 0.01; ***, *P* < 0.001

Since overexpression of *SCARA5* inhibited cell proliferation and increased cell death, we further ascertain the reasons of affecting cell death. Firstly, TE-1 and KYSE150 cells was involved in the absence or presence of several cell death inhibitors. The treatment combined with necrostatin-1 (a potent inhibitor of necroptosis), Z-VAD-FMK (a pan-caspase inhibitor), or 3-Methyladenine (3-MA, a potent inhibitor of autophagy) did not relive the inhibited cell activity in these cells with *SCARA5* overexpression (Fig. 4A), indicating other form of cell death may exist. Hence, the ferrostatin-1(an inhibitor of ferroptosis) and Erastin (an inducer of ferroptosis) were applied into ESCC cells to explore the effect of cell activity. Surprisingly, the cell activity increased in *SCARA5* overexpression group with treatment of ferrostatin-1, but the cell activity was further reduced in *SCARA5* overexpression group with the Erastin (Fig. 4B). Thus, these results indicated that overexpression SCARA5 might induce ferroptosis in ESCC cells.

### Overexpression *SCARA5* causes imbalance of intracellular ROS metabolism

**Fig. 5:**
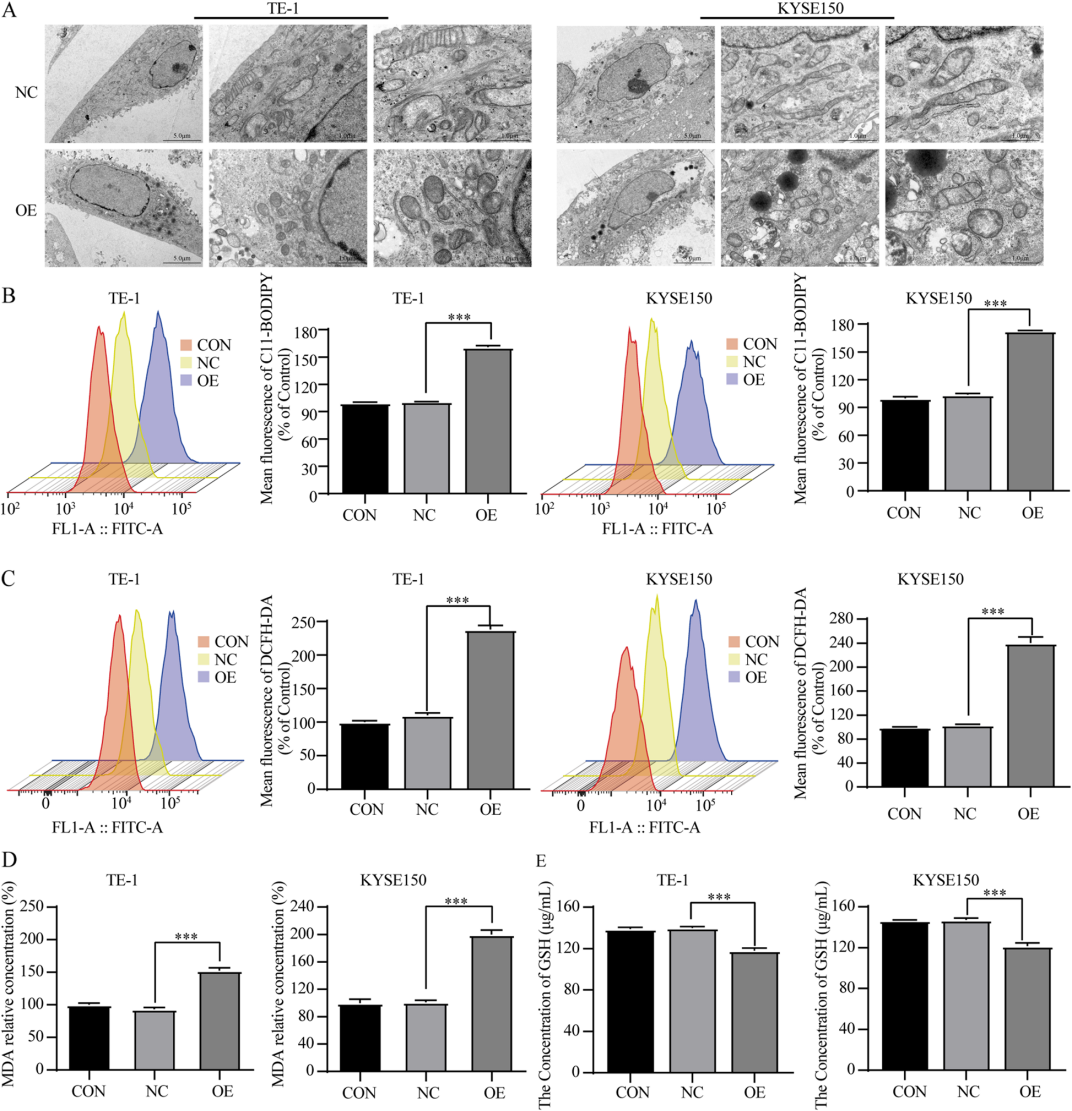
Overexpression SCARA5 induces increased ROS and ferroptosis in ESCC cells. **a** Transmission electron microscopy to detect mitochondrial changes in ESCC cells, scale bar = 5 μm/1 μm. **b** The lipid ROS of ESCC cells was analyzed by a flow cytometer. **c** The intracellular total ROS of ESCC cells was analyzed by a flow cytometer. **d** Intracellular MDA levels in ESCC cells was detected **e** Intracellular GSH level in ESCC cells was detected. ***, *P* < 0.001

To investigate whether ferroptosis was a key determinant in the cell death induced by overexpression *SCARA5*, several ferroptosis events in TE-1 and KYSE150 were detected. Firstly, the morphology of ferroptosis differs from traditional cell death types such as apoptosis and necrosis, and is mainly characterized by reduced mitochondrial volume, decreased cristae, and increased membrane density [19]. Therefore, the mitochondrial morphology was assessed via transmission electron microscopy. The results indicated that the mitochondrial volume and mitochondrial cristae reduced and the mitochondrial membrane density increased in overexpression *SCARA5* group (Fig. 5A). Then, we detected the level of intracellular ROS using a peroxide-sensitive fluorescent probe DCFH-DA and the level of lipid ROS using the peroxide-sensitive fluorescent probe C11-BODIPY. As expected, following overexpression *SCARA5*, the lipid ROS (Fig. 5B) and intracellular ROS (Fig. 5C) accumulation was significantly triggered than of their controls. Moreover, malondialdehyde (MDA), the metabolites of lipid reactive oxygen species, was also showed a significant increase in TE-1 and KYSE150 cells with overexpression *SCARA5*(Fig. 5D). By contrast, GSH level was remarkably decreased, indicating GSH depletion occurred (Fig. 5E). These results confirmed that overexpression of *SCARA5* affected mitochondrial morphological changes and intracellular lipid reactive oxygen species accumulation in ESCC cell, which in turn induced ferroptosis in ESCC cells.

### Overexpression *SCARA5* increased intracellular Fe^2+^

**Fig. 6:**
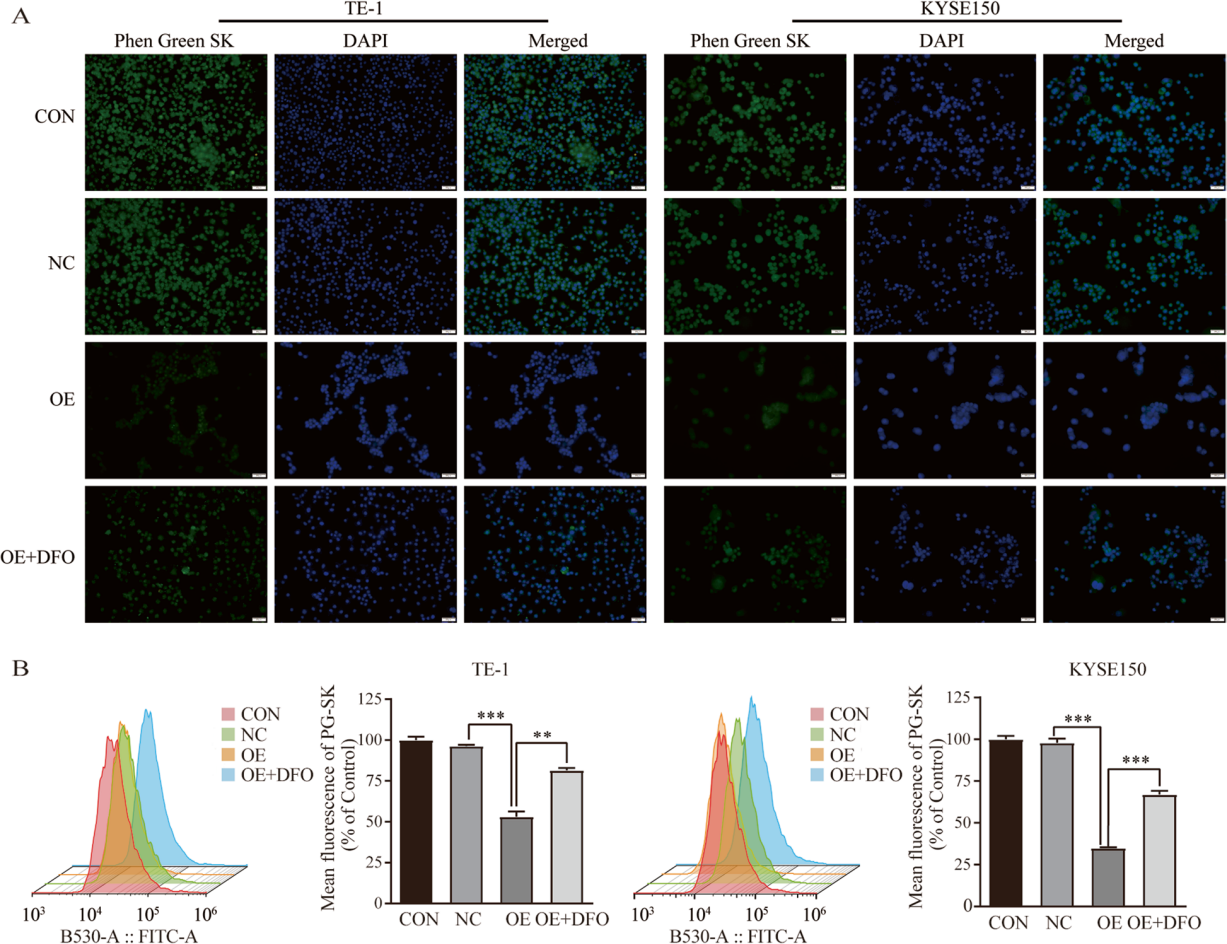
Overexpression SCARA5 induces increased iron ions and ferroptosis in ESCC cells. **a** Immunofluorescence showed the concentration of Fe^2+^ in ESCC cells, scale bar = 50 μm. **b** The Fe.^2+^ in ESCC cells was detected by a flow cytometer. ***, *P* < 0.001

Then, it is well known that iron is the essential reactive element for ferroptosis. Therefore, intracellular chelable iron in TE-1 and KYSE150 cells was further determined using the fluorescent indicator Phen Green SK, the fluorescence of which is quenched by iron. As a result of immunofluorescence, the green fluorescence was reduced in TE-1 and KYSE150 cells overexpressing *SCARA5*, indicated the Fe^2+^ of intra-cellular increased (Fig. 6A). At the same time, the reduction in green fluorescence in overexpressed *SCARA5* TE-1 and KYSE150 cells was almost blocked by the ferroptosis inhibitor deferoxamine (DFO) (Fig. 6A). Furthermore, flow cytometry analysis found that the proportion of Phen Grenn SK-positive cells was decreased in both cell lines with *SCARA5* overexpression, indicating the Fe^2+^ of intracellular accumulation (Fig. 6B). In addition, ferroptosis rescue agents such as DFO restores the proportion of Phen Grenn SK-positive cells in TE-1 and KYSE150 cells overexpressing *SCARA5*(Fig. 6B). Taken together, these findings strongly suggested that overexpression of *SCARA5* triggered Fe^2+^ accumulation in ESCC cells, which in turn induced ferroptosis in ESCC cells.

### SCARA5 induces ferroptosis by combining with Ferritin light chain

**Fig. 7:**
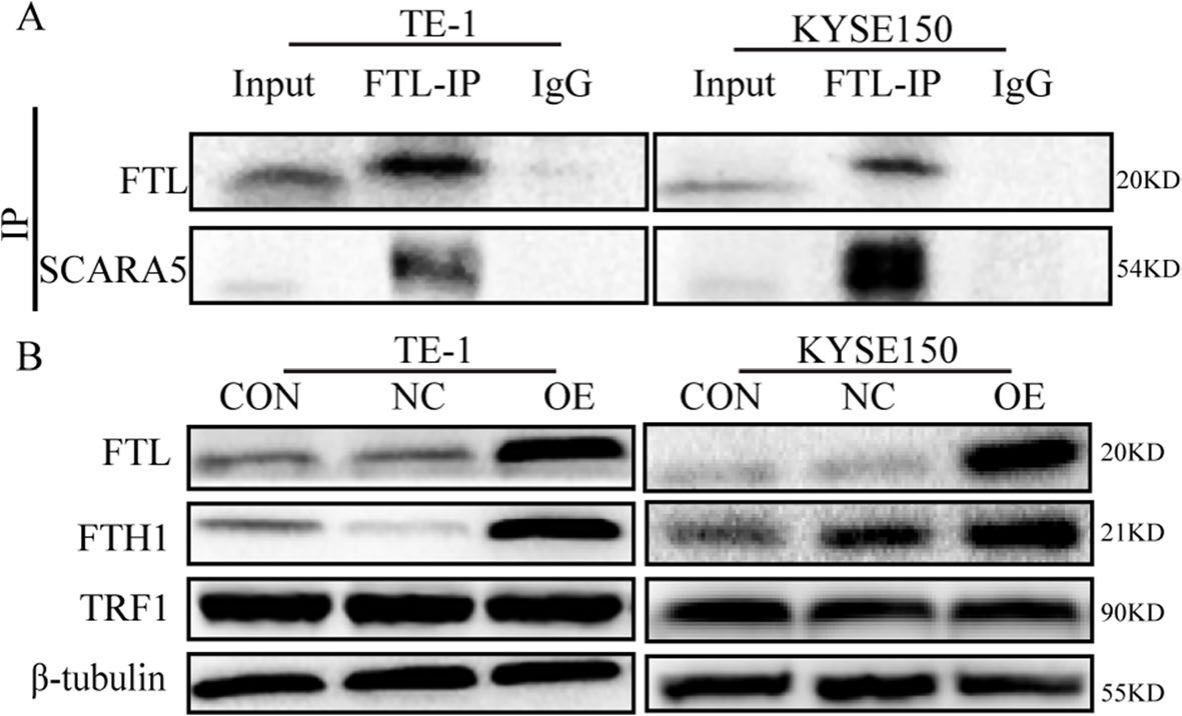
SCARA5 binds to Ferritin light chain to cause ferroptosis. **a** Co-immunoprecipitation analysis of SCARA5 binding to Ferritin light chain. **b** WB analysis of ferroptosis-related protein changes. The original blots/gels are presented in the Supplementary Material 3

To further investigate the inner mechanism by which SCARA5 influences ferroptosis of ESCC, it was that SCARA5 could act as a receptor for ferritin and increase the iron content in unstable iron pools in cells [13]. Therefore, we speculated that there was a similar combine model in ESCC cells. The interaction between SCARA5 and ferritin light chain was explored with Co-immunoprecipitation (Co-IP). The results indicated SCARA5 was combined with ferritin light chain in TE-1 and KYSE150 cells (Fig. 7A). Next, Western blot was further performed to determine the expression of several ferroptosis related proteins. We found that the expression of FTL and FTH1 significantly increased in TE-1 and KYSE150 with overexpression *SCARA5*. Meanwhile, the expression of positive regulator proteins for ferroptosis TFR1 was no changed in all group, suggesting that intracellular Fe^2+^ deliver induced by SCARA5 binding to ferritin light chain is independent of the transferrin-transferrin receptor Fe^2+^ transfer pathway (Fig. 7B). Taken together, these results indicated *SCARA5* induced ferroptosis by combining with ferritin light chain and increased intracellular ferritin light chain.

### *SCARA5* induces ferroptosis in vivo

**Fig. 8:**
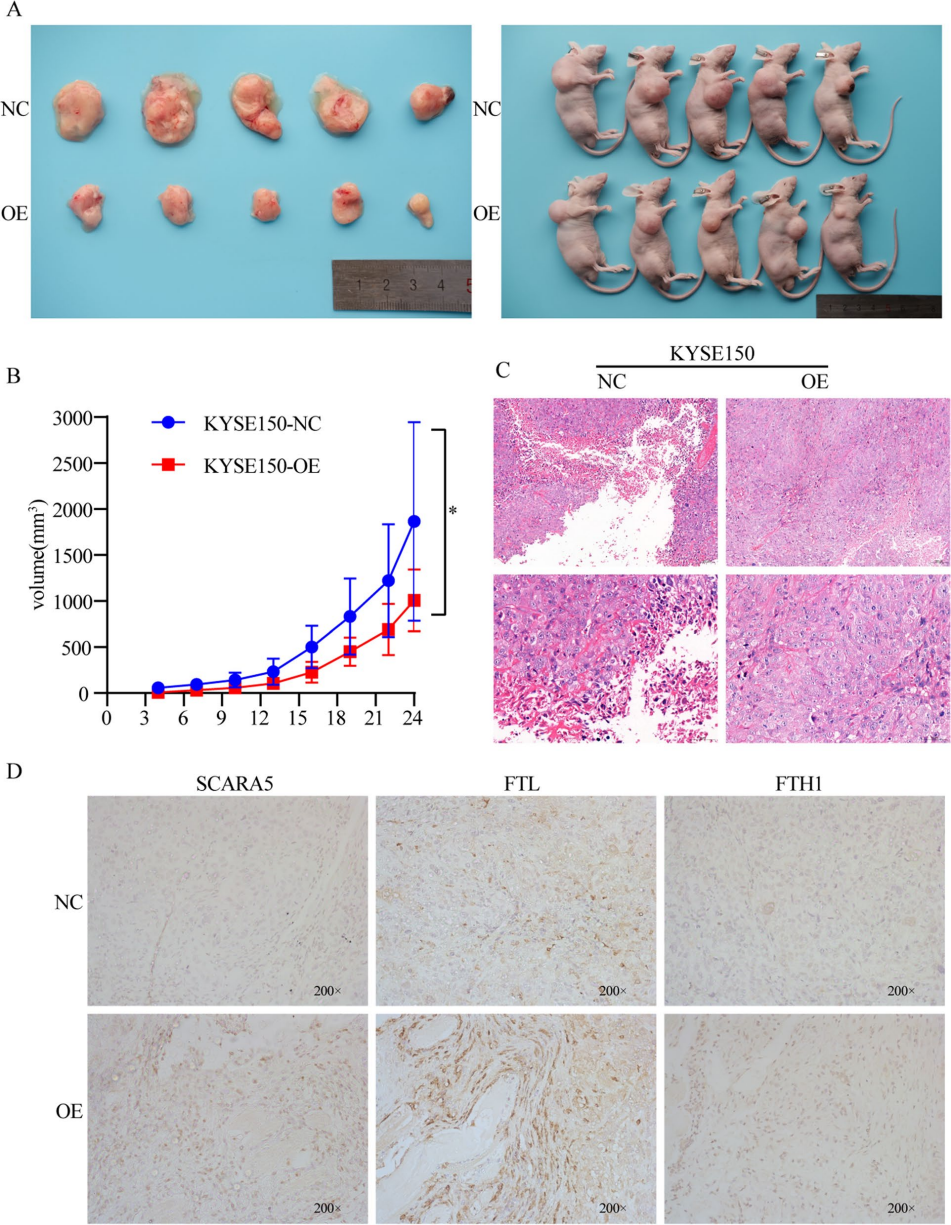
SCARA5 inhibits subcutaneous tumor growth in nude mice. **a** Representative image of tumors from each group of mice. **b** Relative tumor volume with time in each group. **c** HE staining analysis of tumor cell changes, scale bar = 100 μm. **d** Immunohistochemical analysis of the expression of SCARA5/FTL/FTH1 in nude mice tumors. *, *P* < 0.05

We then sought to determine whether SCARA5 could inhibit tumor growth in vivo. The stable *SCARA5*-overexpressing or negative control KYSE150 cells were injected into the left flank of BALB/c nude mice. The result showed that overexpression of SCARA5 significantly reduced the tumor volume (Fig. 8A, B). H&E staining was used to evaluate the morphology of the tumors, and the results indicated tumor necrotic cell decreased in the group of overexpression *SCARA5* (Fig. 8C). Meanwhile, the IHC results showed that the expression of FTL and FTH1 was increased in overexpression *SCARA5* groups (Fig. 8D). Overall, overexpression of *SCARA5* inhibited the growth of subcutaneous tumors in nude mice by increasing intracellular ferritin light chain and inducing ferroptosis in ESCC cells.

## Discussion

Disorder of SCARA5 was believed to be involved in the malignancy of multiple tumors such as, Zheng et al. found that decreasing *SCARA5* promotes Renal Cell Carcinoma proliferation [31]; Wang et al. found that *SCARA5* could suppress the proliferation and promote the apoptosis of retinoblastoma cells by inhibiting the PI3K/AKT pathway [32]. And some researches revealed that *SCARA5* was essential for tumor metastasis and invasion [33–35]. In present study, we found that the expression level of *SCARA5* is significantly lower in ESCC tissues compared with para-carcinoma tissue. Furthermore, CCK-8 and Edu assays indicated that the proliferation ability was significantly reduced in the ESCC cells with SCARA5 overexpression, and the flow cytometry assay of cell cycle revealed that the cell cycle was arrested in G0/G1 phase of the ESCC cells with *SCARA5* overexpression. Meanwhile, the flow cytometry assay revealed that the percentage of cell death was increased in the ESCC cells with *SCARA5* overexpression. Consistent with previous studies, it was also found that overexpression the *SCARA5* obviously inhibited the migration and invasion in ESCC cells. All in all, *SCARA5* could affect the cell proliferation ability, cell death and migration and invasion ability of ESCC cells.

Since overexpression of *SCARA5* increased the cell death of ESCC cells, the cytostatic were used to explored the reason affecting the proliferation activity of ESCC cells, and it was found that apoptosis inhibitors, autophagy inhibitors and necrosis inhibitors did not restore the decrease in cell activity caused by overexpression of *SCARA5*, while ferroptosis inhibitors restored cell viability and the ferroptosis inducer reduced the cell activity in overexpressed *SCARA5* group. The above results suggested that overexpression of *SCARA5* may induce ferroptosis in ESCC cells.

To further validate whether SCARA5 mediated cell death is ferroptosis, we examined several ferroptosis events. Firstly, the mitochondrial changes of the cells were observed by transmission electron microscopy, and it was found that overexpression of *SCARA5* resulted in a decrease in mitochondrial volume, a decrease in mitochondrial cristae, and an increase in mitochondrial membrane density, showing typical ferroptosis morphological changes. Then assaying metabolic indexes revealed that overexpression of *SCARA5* increased intracellular total ROS and lipid ROS, increased the concentration of MDA (a metabolite of lipid reactive oxygen species) and decreased the concentration of GSH (a substrate of glutathione peroxidase metabolism). This suggested that *overexpression* of SCARA5 caused an imbalance of lipid metabolism in ESCC cells, leading to the accumulation of reactive oxygen species and the induction of ferroptosis. In addition, reduced GSH synthesis suggests that overexpression of *SCARA5* may have caused impaired glutathione transport system, which in turn induced ferroptosis. Meanwhile, ferroptosis is an ion-dependent mode of cell death, and we further examined the intracellular Fe^2+^ changes in ESCC cells. By immunofluorescence and flow analysis, we found that overexpression of *SCARA5* resulted in elevated intracellular Fe^2+^ concentration, and the ferroptosis inhibitor DFO can restore the increase of Fe^2+^ in esophageal cancer cells caused by overexpression of *SCARA5*. In summary, overexpression of *SCARA5* caused intracellular reactive oxygen species accumulation and increased intracellular Fe^2+^, which in turn induced ferroptosis in ESCC cells.

**Fig. 9:**
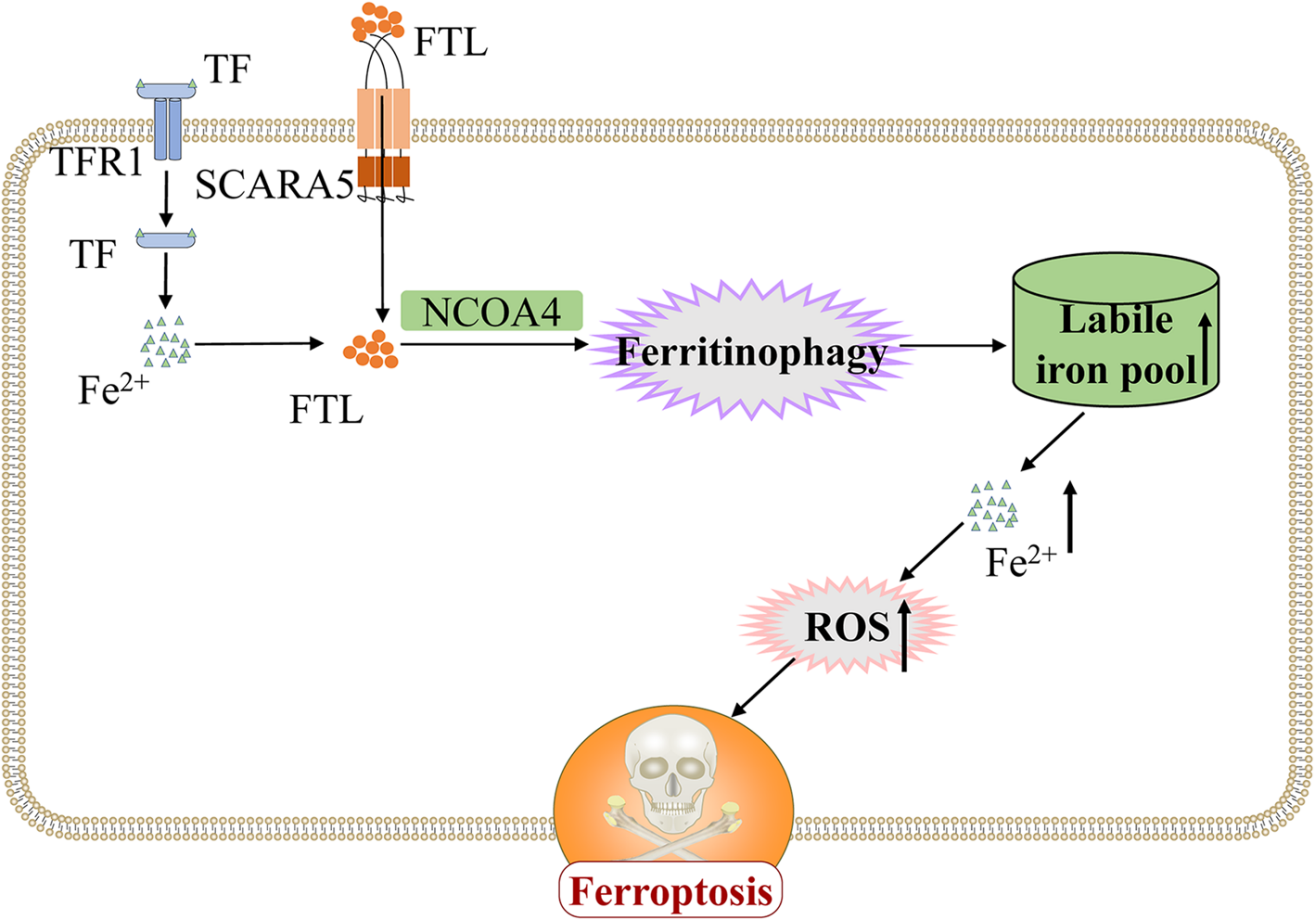
SCARA5 binds to Ferritin light chain to induce ferroptosis in ESCC cells

Studies have reported that the transferrin-transferrin receptor pathway is involved in the occurrence of ferroptosis in a variety of tumors as a classical iron ion delivery pathway [36–38]. As well as, mice with congenital transferrin deficiency die from severe anemia if they are not treated with exogenous transferrin or red blood cell infusions, but still show substantial iron overload in non-hematopoietic tissues such as the liver, kidney, and heart, suggesting a non-transferrin-dependent iron uptake mechanism in these tissues [39]. SCARA5 as a membrane protein, could combine with ferritin light chain and mediate iron delivery of non-transferrin [5, 13, 40]. Ferritin light chain can be delivered to lysosomes for autophagic degradation by the autophagic cargo receptor nuclear receptor coactivator 4 (NCOA4) in cells to release iron ions, which enhances cellular ferroptosis sensitivity by accumulating iron and lipid reactive oxygen species [41, 42]. Therefore, we explore our hypothesis that SCARA5 binds to ferritin light chain, which increased the concentration of ferritin light chain intracellular, and induced ferroptosis. The Co-IP assay demonstrated SCARA5 combines with ferritin light chain, and the WB results also showed that the ferritin light chain increase in the overexpression *SCARA5* groups. In parallel, we examined the transferrin receptor of the classical Fe^2+^ transport pathway, which is often increased when ferroptosis occurs. Our results indicated that overexpression of *SCARA5* does not affect TFR1 expression, suggesting that overexpression of SCARA5-induced ferroptosis pathway is independent of the classical iron ion transport pathway (Fig. 9). Of course, there are still some shortcomings in this study. For example, it is not clear whether the methylation of the SCARA5 promoter causes its down-regulation in ESCC, and it is also unclear what mechanism NCON4 releases Fe^2+^ in ferritin light chain. So, more basic experiments will be needed for further exploration and research.

## Conclusions

In conclusion, the present study found that *SCARA5* exhibited aberrant low expression in ESCC tissues and cell lines, which mainly expressed in the membrane, and which can inhibit proliferation and metastasis of ESCC cells, and induce ferroptosis of ESCC cells. Mechanically, our data indicated that *SCARA5* can induce ferroptosis by combining with ferritin light chain and increasing the accumulation of intracellular reactive oxygen species and Fe^2+^.

## Supplementary Information

**Additional file 1. **

**Additional file 2: Fig. S1.** RT-qPCR shows the differential expression of different genes in esophageal cell lines. The figures show the mRNA expression of SYNM, TNXB, CFD, HSPB6 and PLIN4 in HEEC, HET-1A and ESCC cell lines (* *P*<0.05, *P*<0.01, N.S. vs HEEC and HET-1A).

**Additional file 3. **

**Additional file 4: Supplementary Table 1.** primers sequences.

## Data Availability

“The datasets analyzed during the current study are available in the article and Supplement material”.
