# Supplementary figures and images for: SCARA5 induced ferroptosis to effect ESCC proliferation and metastasis by combining with Ferritin light chain
Source: BMC Cancer. 2022 Dec 13;22:1304. doi: 10.1186/s12885-022-10414-9 (PMC9746006; doi:10.1186/s12885-022-10414-9)

1. Figure 7B-TE-1-TFR1

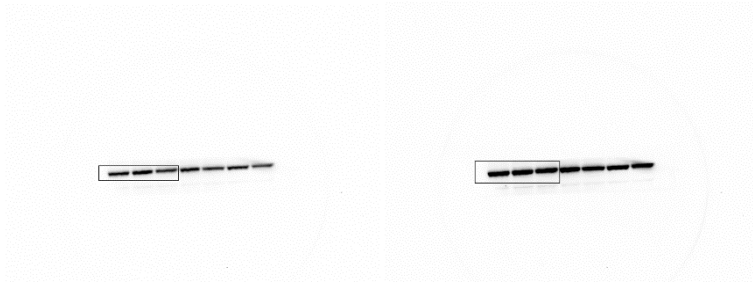

2. Figure 7B-TE-1-tubulin

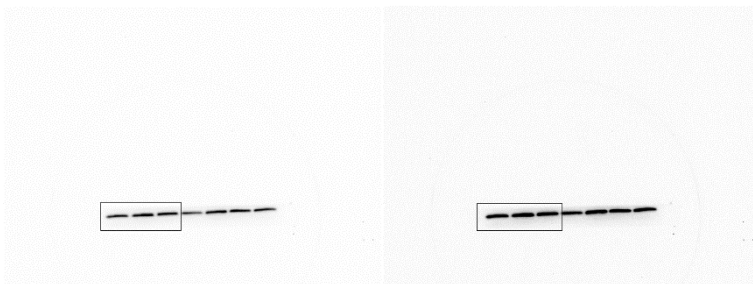

3. Figure 7B-kyse150-TFR1

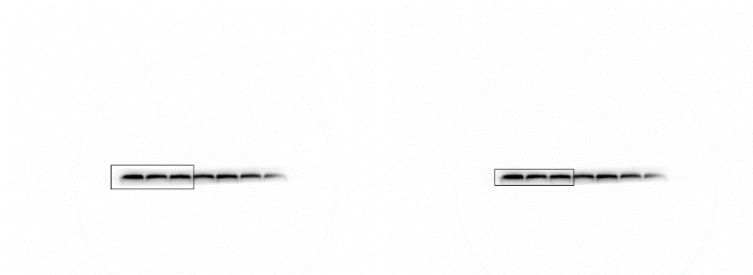

4. Figure 7B-kyse150-tubulin

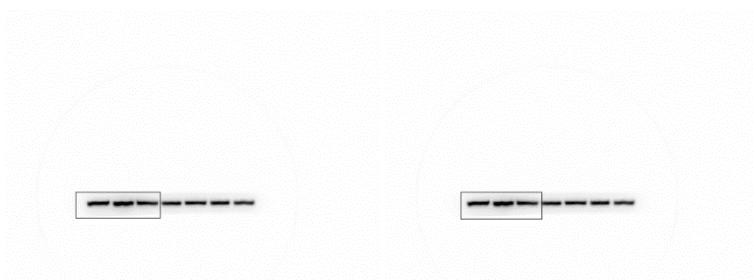

Supplement: Supplementary file 1 — Additional file 1. [file 12885_2022_10414_MOESM1_ESM.pdf]
