# Supplementary material for: SCARA5 induced ferroptosis to effect ESCC proliferation and metastasis by combining with Ferritin light chain
Source: BMC Cancer. 2022 Dec 13;22:1304. doi: 10.1186/s12885-022-10414-9 (PMC9746006; doi:10.1186/s12885-022-10414-9)
Supplement: Supplementary file 2 — Additional file 2: Fig. S1. RT-qPCR shows the differential expression of different genes in esophageal cell lines. The figures show the mRNA expression of SYNM, TNXB, CFD, HSPB6 and PLIN4 in HEEC, HET-1A and ESCC cell lines (* P<0.05, P<0.01, N.S. vs HEEC and HET-1A). [file 12885_2022_10414_MOESM2_ESM.pdf]

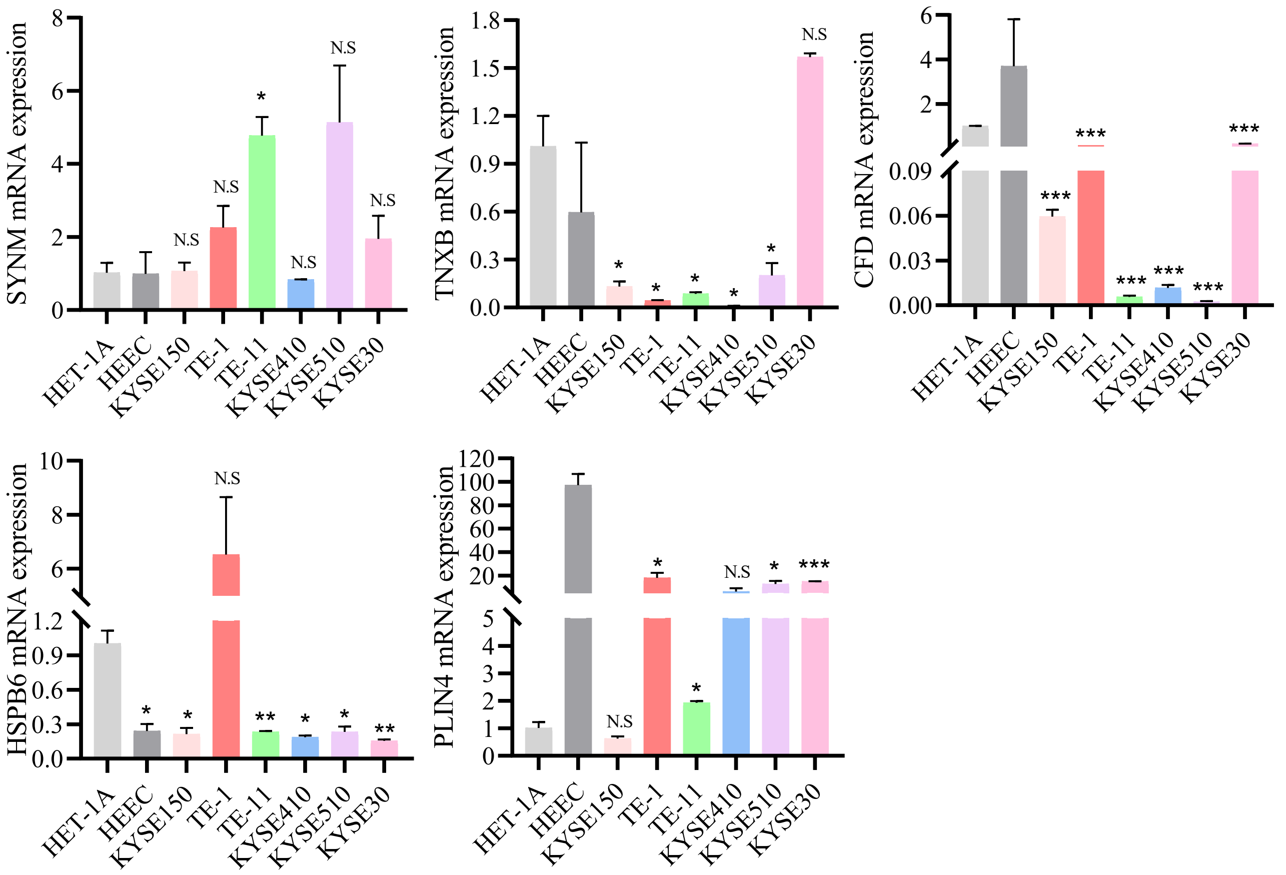

Fig. S1 RT-qPCR shows the differential expression of different genes in esophageal cell lines. The figures show the mRNA expression of SYNM, TNXB, CFD, HSPB6 and PLIN4 in HEEC, HET-1A and ESCC cell lines (* *P* <0.05, *P* <0.01, N.S. vs HEEC and HET-1A).
