# Supplementary material for: SCARA5 induced ferroptosis to effect ESCC proliferation and metastasis by combining with Ferritin light chain
Source: BMC Cancer. 2022 Dec 13;22:1304. doi: 10.1186/s12885-022-10414-9 (PMC9746006; doi:10.1186/s12885-022-10414-9)

Original files of Western blots

Figure 1E SCARA5

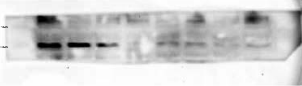

Figure 1E GAPDH

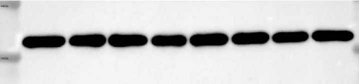

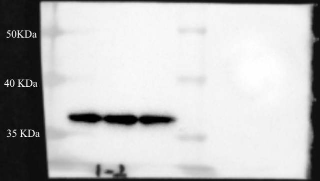

Figure 2A KYSE150 GAPDH

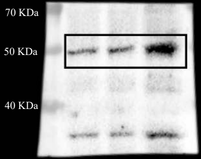

Figure 2A KYSE150 SCARA5

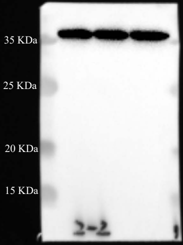

Figure 2A TE-1 GAPDH

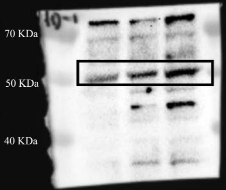

Figure 2A TE-1 SCARA5

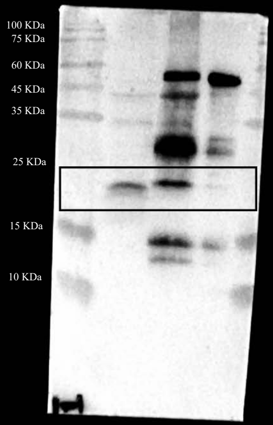

Figure 7A TE-1 FTL

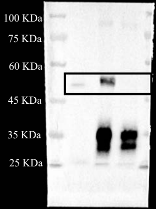

Figure 7A TE-1 SCARA5

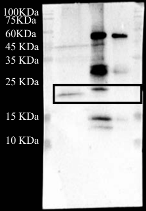

Figure 7A KYSE150 FTL

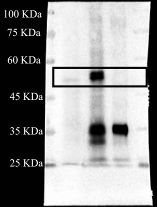

Figure 7A KYSE150 SCARA5

Figure 7B TE-1 FTH1

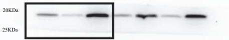

Figure 7B TE-1 FTL

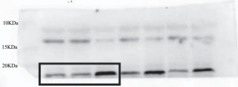

Figure 7B TE-1 TRF1

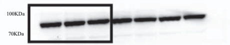

Figure 7B TE-1
tubulin

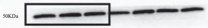

Figure 7B KYSE150
FTH1

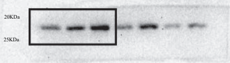

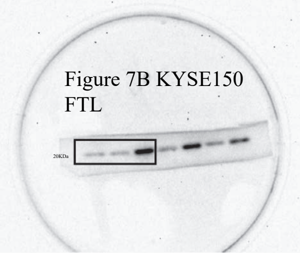

Figure 7B KYSE150
FTL
20KDa

Figure 7B KYSE150
TFR1

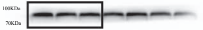

Figure 7B KYSE150 tubulin

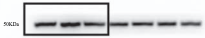

Supplement: Supplementary file 3 — Additional file 3. [file 12885_2022_10414_MOESM3_ESM.pdf]
