# Supplementary material for: SCARA5 induced ferroptosis to effect ESCC proliferation and metastasis by combining with Ferritin light chain
Source: BMC Cancer. 2022 Dec 13;22:1304. doi: 10.1186/s12885-022-10414-9 (PMC9746006; doi:10.1186/s12885-022-10414-9)
Supplement: Supplementary file 4 — Additional file 4: Supplementary Table 1. primers sequences. [file 12885_2022_10414_MOESM4_ESM.pdf]

Supplementary Table 1 primers sequences

	Forward	Reverse
SCARA5	CTGGATGGATGACGTTGCCT	GTCTGTTGCATGTCACGCTG
GAPDH	TGCACCACCAACTGCTTAGC	GGCATGGACTGTGGTCATGAG
SYNM	AAGTGGTGGAGGTAAGTGCG	TGGACAGTGGACTTTGACCG
TNXB	AGCACCATCTTCCTCAACGG	ATGGGCATAGTCCTCCCAGT
CFD	GCTACAGCTGTCGGAGAAGG	CGTGGTTGACTATGCCCCAG
HSPB6	ACAGTCTACAACTGCCCCCT	GGGTGTTCTAGTTGGCCTGG
PLIN4	GCACATAGCTCGGCGAGAG	CGGACACCATCTTTTCCGAAG
